# Supplementary material for: Impact of Water Exposure and Temperature Changes on Skin Barrier Function
Source: J Clin Med. 2022 Jan 7;11(2):298. doi: 10.3390/jcm11020298 (PMC8778033; doi:10.3390/jcm11020298)
Supplement: Supplementary file 1 [file jcm-11-00298-s001.zip › jcm-1484538-supplementary.pdf]

Table S1. Changes in skin barrier function after cold and hot water exposure

	**Basal**	**Cold water**	**Hot water**	$\mathbf{p^{B\text{-}C}}$	$\mathbf{p^{B\text{-}H}}$	$\mathbf{p^{C\text{-}H}}$
**Temperature (ºC)**	30.18 (1.84 SD)	23.49 (2.59 SD)	32.60 (1.21 SD)	<0.001	<0.001	<0.001
**pH**	6.33 (0.37 SD)	6.62 (0.30 SD)	6.65 (0.29 SD)	<0.001	<0.001	0.369
**Erythema (AU)**	249.45 (57.25 SD)	253.63 (56.78 SD)	286.34 (55.88 SD)	0.603	<0.001	<0.001
**TEWL ($g{\cdot}h^{-1}{\cdot}m^{-2}$)**	25.75 (12.40 SD)	34.96 (8.54 SD)	58.58 (10.25 SD)	<0.001	<0.001	<0.001
**SCH (AU)**	46.69 (18.73 SD)	50.55 (14.90 SD)	44.50 (13.91 SD)	0.040	0.324	0.004

AU, arbitrary units; SCH, stratum corneum hydration; TEWL, transepidermal water loss.

Data are expressed as means (standard deviations, SD).

$^{B\text{-}C}$ *p*-value after using Student’s *t*-test for paired samples to compare skin barrier function between baseline and after cold water exposure.

$^{B\text{-}H}$ *p*-value after using Student’s *t*-test for paired samples to compare skin barrier function between baseline and after hot water exposure.

$^{C\text{-}H}$ *p*-value after using Student’s *t*-test for paired samples to compare skin barrier function after cold and hot water exposure.

Table S2. Changes in skin barrier function after TempTest

	Basal	4ºC TempTest	44ºC TempTest	p^{B-C}	p^{B-H}	p^{C-H}
**Temperature (ºC)**	30.76 (1.24 SD)	25.35 (2.18SD)	32.45 (1.45 SD)	<0.001	<0.001	<0.001
**pH**	6.27 (0.37 SD)	6.28 (0.36 SD)	6.25 (0.36 SD)	0.435	0.419	0.132
**Erythema (AU)**	210.45 (60.85 SD)	209.07 (64.50 SD)	227.79 (60.20 SD)	0.847	0.001	0.017
**TEWL ($g \cdot h^{-1} \cdot m^{-2}$)**	7.99 (3.39 SD)	8.74 (3.58 SD)	9.98 (3.86 SD)	0.145	<0.001	0.003
**SCH (AU)**	37.77 (12.94 SD)	39.51 (11.60 SD)	38.01 (11.95 SD)	0.077	0.847	0.121

AU, arbitrary units; SCH, stratum corneum hydration; TEWL, transepidermal water loss.

Data are expressed as means (standard deviations, SD).

$^{B-C}$ *p*-value after using Student's *t*-test for paired samples to compare skin barrier function between baseline and after 4ºC Temptest exposure.

$^{B-H}$ *p*-value after using Student's *t*-test for paired samples to compare skin barrier function between baseline and after 44ºC Temptest exposure.

$^{C-H}$ *p*-value after using Student's *t*-test for paired samples to compare skin barrier function after 4ºC and 44ºC Tempstest exposure.
